# Supplementary material for: Capillary flow experiments for thermodynamic and kinetic characterization of protein liquid-liquid phase separation
Source: Nat Commun. 2021 Dec 15;12:7289. doi: 10.1038/s41467-021-27433-y (PMC8674230; doi:10.1038/s41467-021-27433-y)
Supplement: Supplementary file 1 — Supplementary Information [file 41467_2021_27433_MOESM1_ESM.pdf]

Supplementary Information

Capillary flow experiments for thermodynamic and kinetic characterization of protein liquid-liquid phase separation

Emil G. P. Stender[1,§], Soumik Ray[1,§], Rasmus K. Norrild[1,§], Jacob Aunstrup Larsen[1], Daniel Petersen[2], Azad Farzadfard[1], Céline Galvagnion[2], Henrik Jensen[3] and Alexander K. Buell*[1]

(1) E.G.P. Stender, S. Ray, R. K Norrild, J. A. Larsen, A. Farzadfard, A. K. Buell
Department of Biotechnology and Biomedicine
Technical University of Denmark – DTU
Søltofts Plads building 227
2800 Kgs. Lyngby
Denmark

(2) D. Petersen, C. Galvagnion
Department of Pharmacology and Drug Design
Universitetsparken 2
2100 Copenhagen
Denmark

(3) CSO H. Jensen
FIDA Biosystems Aps
Fruebjergvej 3
2100 Copenhagen
Denmark

*Correspondence: Alexander K. Buell, E-mail: alebu@dtu.dk

[§] These authors contributed equally.

Table of Contents

Supplementary Figures 1-13	Pages 3-15
Supplementary Table 1	Pages 16-19

Supplementary Figures

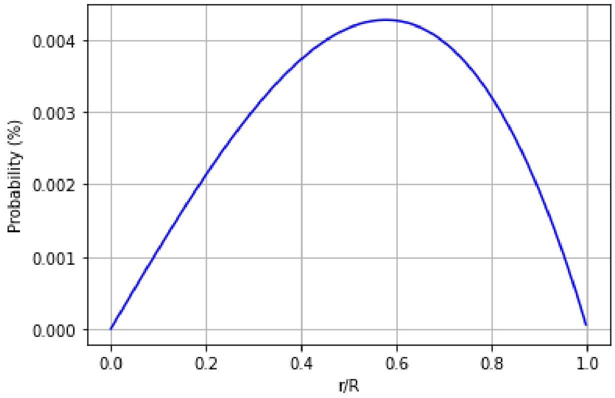

Supplementary Figure 1. Particle flux as a function of radius from center of the capillary, assuming random particle distribution.

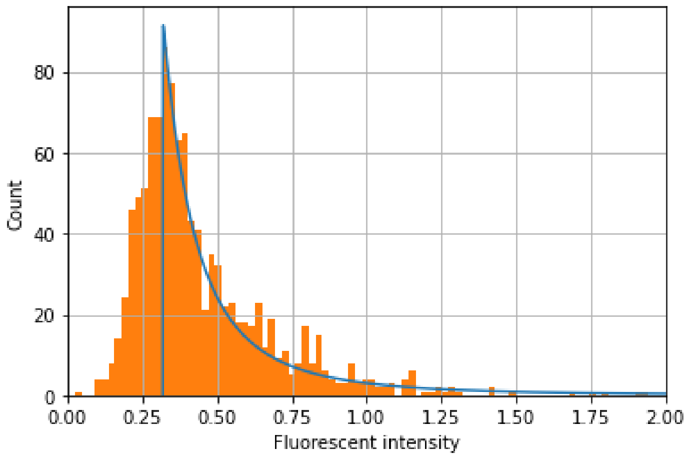

Supplementary Figure 2. Expected signal distribution (blue line) overlaid the 1 μm polystyrene sphere calibration data (histogram).

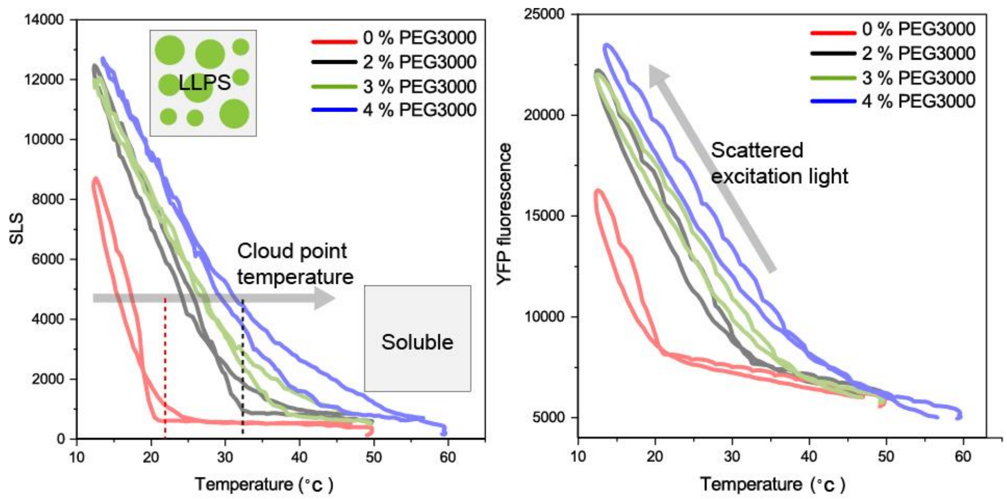

Supplementary Figure 3. Cloud point determination, LLPS reversibility and the influence of PEG3000. (***Left panel***) Thermal ramp of 50 µM Ddx4n1 and 0-4 % PEG3000 monitored by static light scattering intensity. (***Right panel***) Thermal ramp of 50 µM Ddx4n1 and 0-4 % PEG3000 monitored by YFP fluorescence 0 % (Red), 2 % (black), 3 % (green), and 4 % (blue). The cloud point increases as a function of PEG3000 concentration and the process is reversible. Increase in YFP fluorescence upon passing the cloud point is due to the ProbeDrum instrument being without a detector filter in order to be able to record multiple channels at once, which leads to the detection of scattered excitation light. When no LLPS is present in the samples at 50 °C there is no difference in YFP fluorescence in the samples with different PEG concentrations, indicating, that there is no quenching of YFP signal by addition of PEG3000. The experiments are carried out three times with similar observations. Source data are provided as a Source Data file.

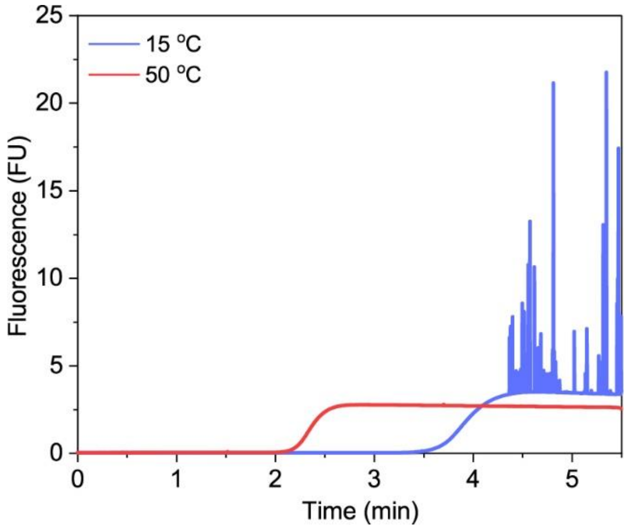

Supplementary Figure 4. Ddx4n1 liquid droplets dissolve above cloud point temperature in Capflex: Appearance of spikes in the fluorescence signal confirms LLPS of 140 µM Ddx4n1 (500 nM YFP Ddx4n1 as reporter) at 15 ºC (blue trace). The same sample when heated to 50 ºC shows no signal spikes confirming the reversible nature of liquid Ddx4n1 droplets. Both the sample tray and capillary temperatures are kept at identical temperatures during each measurement. Representative Capflex traces are reported. The experiment was performed two times with similar observations. Source data are provided as a Source Data file.

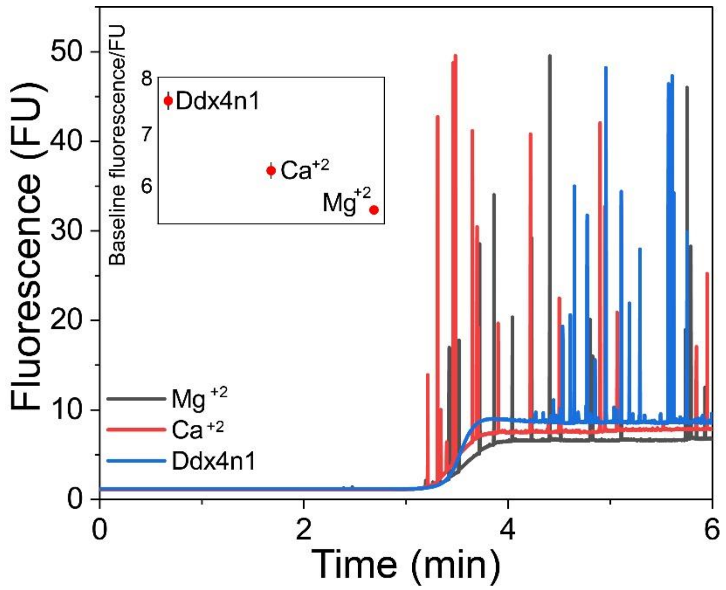

Supplementary Figure 5. Effect of Mg^{+2} on the phase separation behavior of Ddx4n1: Capflex traces for 100 µM Ddx4n1 (with 500 nM YFP Ddx4n1) in the absence (***blue***) and in the presence of 10 mM Ca^{+2} (***red***) and Mg^{+2} (***black***) is shown. The sample tray temperature was maintained at 50 ºC and the capillary temperature was kept at 15 ºC for this experiment. The inset represents relative decrease of the baseline concentration in the presence of both Ca^{+2} and Mg^{+2}. Representative Capflex traces are shown. Source data are provided as a Source Data file.

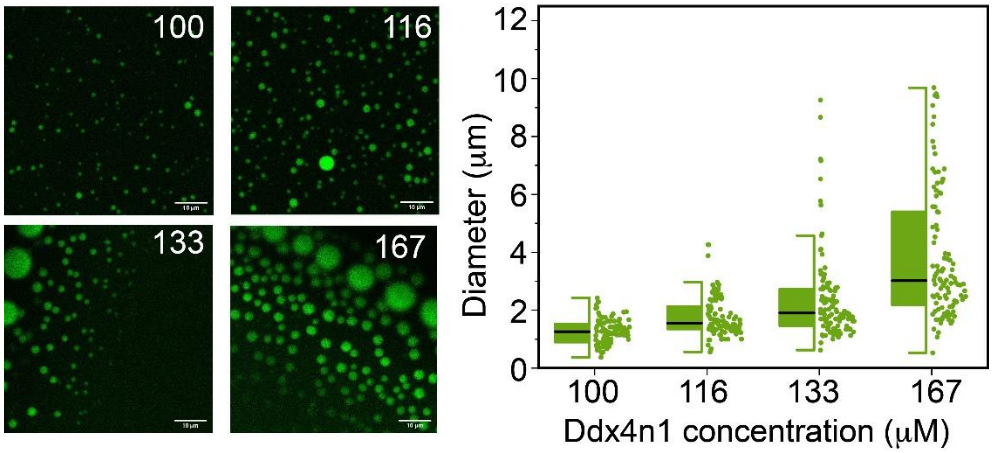

Supplementary Figure 6. Evolution of droplet size distribution of Ddx4n1 with total protein concentration: Representative fluorescence microscopic images of Ddx4n1 droplets are shown for 100, 116, 133 and 167 µM total protein concentrations. The right panel shows the distribution of the droplet sizes (**Supplementary Table 8**) for 100, 116, 133 and 167 µM total Ddx4n1 concentrations for 100 liquid droplets per sample. n=2 independent experiments. The values represent individual droplet diameters (size) and the black line represents the median of the distribution. Source data are provided as a Source Data file.

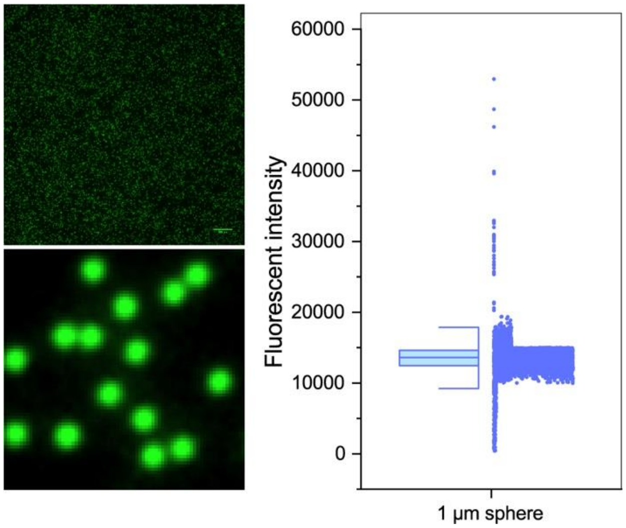

Supplementary Figure 7. (***Left panel***) Representative Fluorescence microscopy image of the 1 μm polystyrene spheres. (Bottom panel) Magnified fluorescence microscopy image of the 1 μm polystyrene spheres is shown for better visualization. (***Right panel***) fluorescence intensity distribution from the microscopy image is plotted. The fluorescence intensity distribution of the 1 μm calibration spheres (**Supplementary Table 8**) is narrow indicating that spheres have a homogeneous fluorescence intensity. However, a significant number of particles with lower intensity are observed, potentially explaining the tail of the Capflex peak intensity distribution when these calibration spheres are measured (**Supplementary Figure 2**). The experiments are carried out 2 times with similar observations. Source data are provided as a Source Data file.

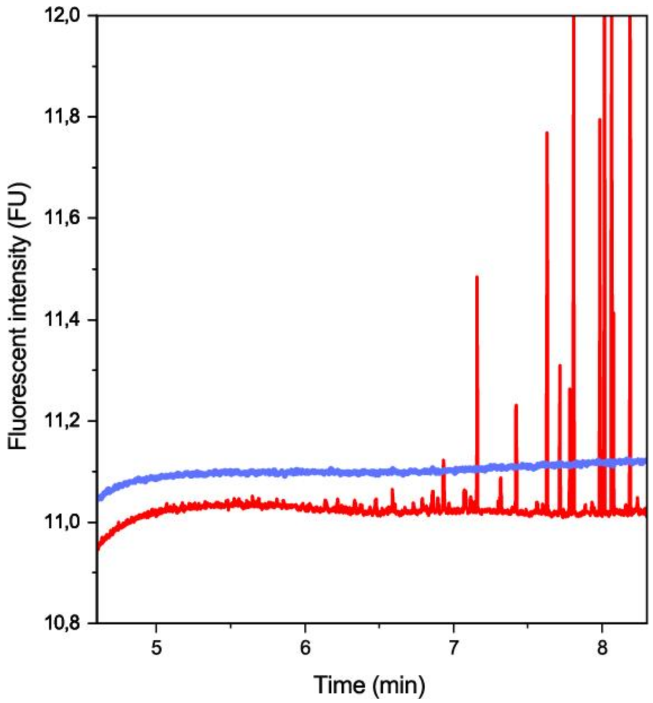

Supplementary Figure 8. Capflex of 112 µM Ddx4n1 with 6 µM total ssDNA, 30 nM L-DNA (red) and in the absence of Ddx4n1, with 4 µM total ssDNA, 20 nM L-DNA (blue) is shown. The ssDNA is incapable of undergoing LLPS on its own and therefore the blue line corresponds to the free signal of 4 µM ssDNA and gives an impression of the very low noise level when no LLPS has occurred. The red line corresponds to 112 µM Ddx4 with 6 µM total ssDNA. The baseline is noticeably noisier compared to the 4 µM ssDNA demonstrating that very small droplets are likely present, that cannot easily be distinguished from baseline noise. The experiments are carried out 2 times with similar observations. Source data are provided as a Source Data file.

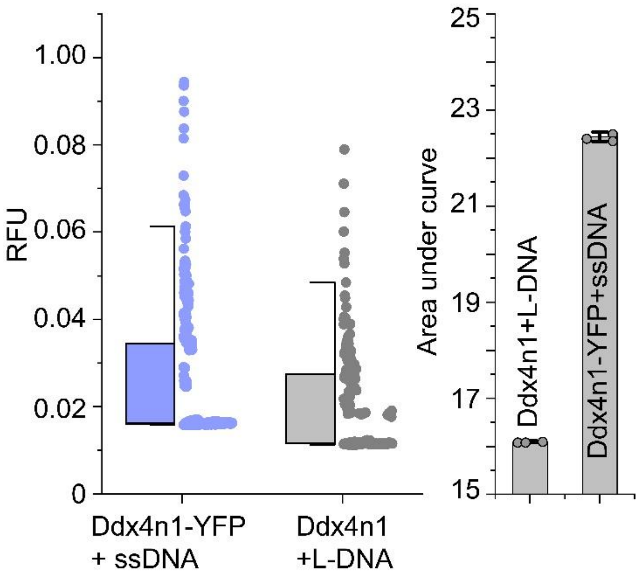

Supplementary Figure 9. Data used to calculate the relative co-partitioning of ssDNA and protein. Integrated signal spikes for 3 data sets with Ddx4n1-YFP + non-labeled ssDNA and non-labeled Ddx4n1 + L-DNA (***left panel***) are shown (**Supplementary Table 8**). Area under the curve with integrated baseline is shown for Ddx4n1-YFP + non-labeled ssDNA and non-labeled Ddx4n1 + L-DNA (***right panel***). Data represents mean ± S.D. for n=3 independent experiments. Source data are provided as a Source Data file.

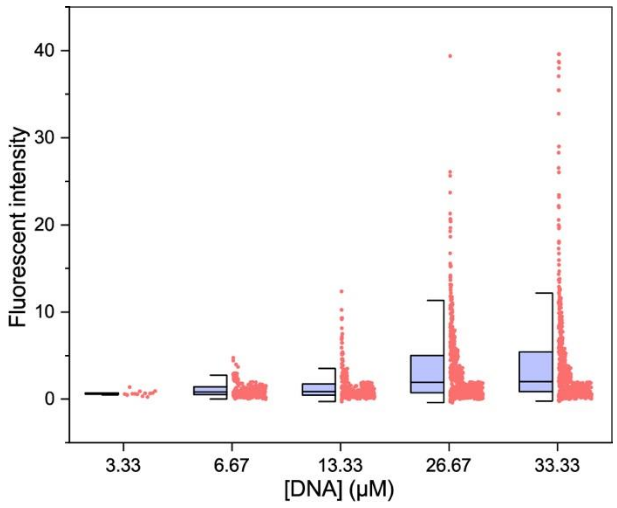

Supplementary Figure 10. Droplet size distribution of RP$_3$/ssDNA coacervation: The droplet intensity is normalized to the baseline to compensate for increased presence of L-DNA. Representative data is shown (**Supplementary Table 8**). The experiment is carried out twice with similar observations. Source data are provided as a Source Data file.

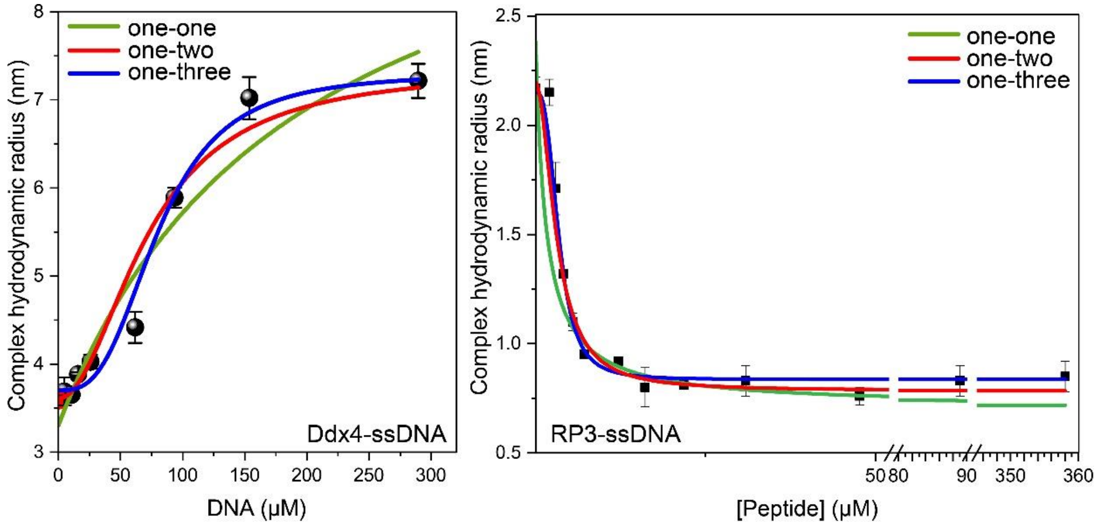

Supplementary Figure 11. Analysis of Ddx4n1 and RP$_3$ binding to ssDNA: A one to one (green), two (red) or three (blue) model assuming independent binding sites fitted to the binding data with ssDNA for Ddx4n1 (***left panel***) RP$_3$ peptide (***right panel***). A one-to-one model fits both datasets poorly. As there is a large difference in the relative size of the binding partners in both cases, a multivalent model seems reasonable to assume. There is no significant difference between the dissociation constant yielded by the one-to-two and the one-to-three binding models, and hence we applied the 1:2 binding model in both cases. Data represents mean ± SD for n=3 independent experiments. Source data are provided as a Source Data file.

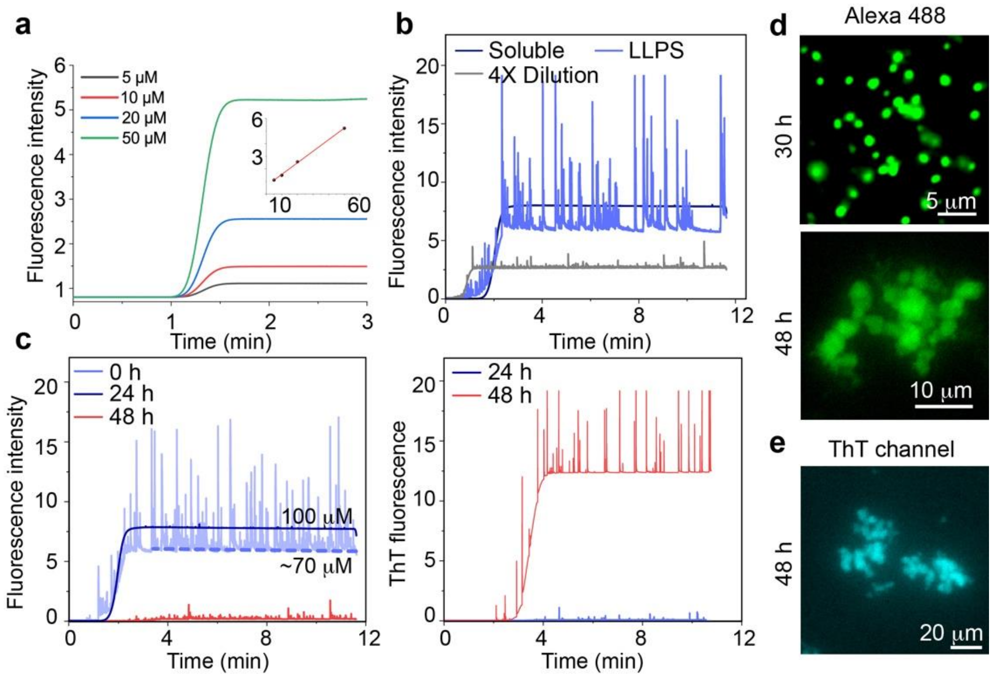

Supplementary Figure 12. N-acetylated α-Syn can co-partition inside WT α-Syn droplets without altering LLPS behaviour: a. Standard curve calibration of the baseline using 5, 10, 20 and 50 µM soluble α-Syn (with 5 nM Alexa488-N-acetylated α-Syn A140C) is shown. **b.** Signal spikes corresponding to droplets containing Alexa488-N-acetylated α-Syn are observed when 100 µM α-Syn is phase separated in the presence of 20% (w/v) PEG6000 observed after 24 h of incubation at 37 °C. The dilute phase concentration decreases from 100 µM to ~70 µM after 24 h. The fluorescence baseline further decreases when the LLPS sample is diluted with buffer and the signal spikes are strongly decrease in frequency and intensity, indicating re-dissolution of the droplets. **c.** (***Left panel)*** LLPS behavior of WT α-Syn spiked with Alexa488-N-acetylated α-Syn A140C using Capflex is shown. The dilute phase concentration decreases from 100 µM to ~70 µM with signal spikes after 24 h in the Alexa488 channel. After 48 h, the dilute phase concentration, as inferred from Alexa488 fluorescence in the background, is found to have dropped by more than one order of magnitude. (***Right panel***) The ThT channel does not show a significant fluorescence signal at 24 h. However, after 48 h, ThT fluorescence shows a substantial increase in the baseline and also features spikes, indicating ThT positive aggregation of α-Syn. **d.** Representative fluorescence microscopic images of the droplets at 24 h, 36 h and 48 h are shown. The data indicates successful partitioning of Alexa488-N-acetylated α-Syn A140C inside the droplets **e.** Representative fluorescence microscopic images of the droplets at 48 h are shown with a ThT specific fluorescence channel. The droplets show successful ThT binding, indicating the presence of ThT positive, amyloid aggregates inside them. The experiments (a-d) are carried out three times with similar observations. Source data are provided as a Source Data file.

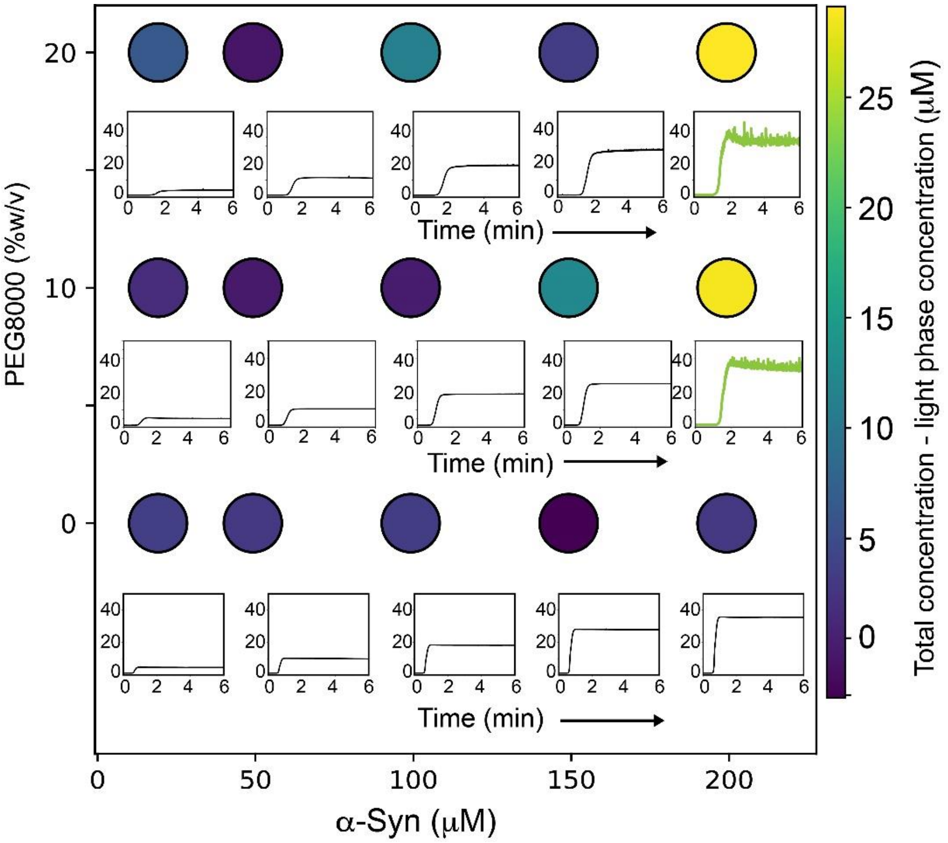

Supplementary Figure 13. Two dimensional LLPS screen of α-Syn at HTP: Samples of 20, 50, 100, 150 and 200 µM α-Syn (in 20 mM PBS, pH 7.4) in the presence of increasing (0, 10 and 20% (w/v) PEG8000 concentration are analyzed with Capflex. The solutions are spiked with 50 nM (0.025%) Alexa488 maleimide labeled A140C α-Syn. 200 µM α-Syn in the presence of 10 and 20% (w/v) PEG8000 shows clear signs of LLPS as confirmed by the appearance of spikes in the fluorescence signal and a substantial decrease in the baseline fluorescence signal (shown as insets). The mean dilute phase equivalent concentration is represented with color codes (***right***) and quantified from the baseline fluorescence intensities for individual samples (represented as circles) for n=2 independent observations. Representative Capflex traces are shown. The traces are indicated in black when no LLPS was observed and green when LLPS was observed. Source data are provided as a Source Data file.

Supplementary Table 1: Description of the box whisker plots used to analyze peak intensity/droplet size distribution: The upper and lower quartile, maxima, minima, and center/median of the box whisker plots and their respective percentiles are described in this table for **Figure 1g, Figure 1i, Figure 2a-b** (***right panels***), and **Supplementary Figure 6, 7, 9** and **10.** The maximum outlier value is also reported for each distribution wherever applicable. The data is plotted in the range of 25 to 75 percentile. The whisker coefficient was taken as (1.5IQR). The values are represented in fluorescence intensity units (FU).

Figure panels	Description	Upper quartile in FU (percentile (%))	Lower quartile in FU (percentile (%))	Center/median in FU (percentile (%))	Minima-maxima of the whiskers (FU)	Maximum outlier (FU)
**Figure 1g**	100 µM Ddx4n1	7.4 (75%)	6.1 (25%)	6.7 (50%)	5.0-8.2	11.0
	116 µM Ddx4n1	15.2 (75%)	7.5 (25%)	10.7 (50%)	5.0-26.7	43.2
	133 µM Ddx4n1	32.2 (75%)	10.3 (25%)	18.5 (50%)	4.7-49.1	n/a
	167 µM Ddx4n1	45.5 (75%)	8.3 (25%)	17.2 (50%)	4.8-49.1	n/a
	1 mM Ca^{+2}	5.1 (75%)	4.9 (25%)	5.0 (50%)	4.7-5.3	5.9
	2 mM Ca^{+2}	5.9 (75%)	4.4 (25%)	4.9 (50%)	3.8-8.1	11.5
	3 mM Ca^{+2}	7.1 (75%)	4.2 (25%)	5.1 (50%)	3.5-11.4	40.7
	4 mM Ca^{+2}	10.1 (75%)	5.2 (25%)	6.9 (50%)	3.1-17.3	28.6
	5 mM Ca^{+2}	17.2 (75%)	7.0 (25%)	11.1 (50%)	3.0-31.6	46.4
	10 mM Ca^{+2}	18.3 (75%)	7.4 (25%)	10.5 (50%)	2.7-34.4	47.2
	2% PEG3000	2.6 (75%)	2.0 (25%)	2.3 (50%)	1.9-3.3	4.8
	3% PEG3000	10.6 (75%)	4.0 (25%)	6.6 (50%)	1.6-16.2	23.3
	4% PEG3000	16.2 (75%)	7.7 (25%)	12.0 (50%)	1.8-23.8	32.0
	5% PEG3000	14.1 (75%)	8.4 (25%)	10.7 (50%)	1.2-18.0	48.9

	6% PEG3000	17.4 (75%)	8.3 (25%)	11.9 (50%)	1.0-30.6	46.4
	7% PEG3000	29.9 (75%)	11.2 (25%)	16.0 (50%)	0.8-48.9	n/a
**Figure 1i**	1 µm spheres	0.5 (75%)	0.3 (25%)	0.4 (50%)	0.02-0.9	2.25
	6 µm spheres	43.3 (75%)	32.0 (25%)	38.6 (50%)	17.1-47.4	n/a
**Figure 2a**	0 µM DNA	23.3	8.6	15.1	4.1-45.0	48.0
	1 µM DNA	18.0	7.7	12.3	5.1-32.9	48.0
	2 µM DNA	29.5	10.9	17.4	5.1-48.1	n/a
	3 µM DNA	28.7	11.6	19.8	5.1-48.0	n/a
	5 µM DNA	12.2	7.1	8.5	5.7-19.5	43.9
	6 µM DNA	15.2	7.0	9.8	5.6-26.1	36.5
**Figure 2b**	1 µM DNA	8.9	4.7	6.7	2.2-15.0	17.4
	2 µM DNA	12.0	6.5	8.2	4.5-19.5	23.4
	3 µM DNA	11.5	6.9	8.8	5.2-17.8	20.6
	4 µM DNA	12.2	8.4	10.7	7.5-16.4	19.1
	6 µM DNA	10.2	9.6	9.8	9.6-10.5	n/a
**Supplementary Figure 6**	100 µM Ddx4n1	1.5	0.9	1.2	0.3-2.4	n/a
	116 µM Ddx4n1	2.1	1.3	1.5	0.5-2.9	4.3

	133 µM Ddx4n1	2.7	1.4	1.9	0.6-4.5	9.3
	167 µM Ddx4n1	5.3	2.1	3.0	0.5-9.7	n/a
**Supplementary Figure 7**	1 µm sphere	14648	12479	13601	9228-17860	52947
**Supplementary Figure 9**	Ddx4n1-YFP + non-labeled ssDNA	0.03	0.016	0.016	0.01-0.06	0.09
	Ddx4n1 + L-DNA	0.03	0.01	0.01	0.01-0.05	0.08
**Supplementary Figure 10**	3.33 µM DNA	0.77	0.5	0.6	0.4-0.77	1.4
	6.67 µM DNA	1.4	0.5	0.8	0.005-2.7	4.8
	13.33 µM DNA	1.7	0.4	0.8	0-3.5	12.3
	26.67 µM DNA	5.0	0.7	1.9	0-11.3	39.3
	33.33 µM DNA	5.4	0.8	2.0	0.2-12.1	39.6
