# Supplementary material for: Capillary flow experiments for thermodynamic and kinetic characterization of protein liquid-liquid phase separation
Source: Nat Commun. 2021 Dec 15;12:7289. doi: 10.1038/s41467-021-27433-y (PMC8674230; doi:10.1038/s41467-021-27433-y)
Supplement: Supplementary file 2 — Reporting Summary [file 41467_2021_27433_MOESM2_ESM.pdf]

nature portfolio

Corresponding author(s): Alexander K. Buell

Last updated by author(s): Nov 8, 2021

Reporting Summary

Nature Portfolio wishes to improve the reproducibility of the work that we publish. This form provides structure for consistency and transparency in reporting. For further information on Nature Portfolio policies, see our Editorial Policies and the Editorial Policy Checklist.

Statistics

For all statistical analyses, confirm that the following items are present in the figure legend, table legend, main text, or Methods section.

n/a	Confirmed	
[ ]	[x]	The exact sample size (n) for each experimental group/condition, given as a discrete number and unit of measurement
[ ]	[x]	A statement on whether measurements were taken from distinct samples or whether the same sample was measured repeatedly
[x]	[ ]	The statistical test(s) used AND whether they are one- or two-sided<br>*Only common tests should be described solely by name; describe more complex techniques in the Methods section.*
[x]	[ ]	A description of all covariates tested
[x]	[ ]	A description of any assumptions or corrections, such as tests of normality and adjustment for multiple comparisons
[ ]	[x]	A full description of the statistical parameters including central tendency (e.g. means) or other basic estimates (e.g. regression coefficient) AND variation (e.g. standard deviation) or associated estimates of uncertainty (e.g. confidence intervals)
[x]	[ ]	For null hypothesis testing, the test statistic (e.g. F, t, r) with confidence intervals, effect sizes, degrees of freedom and P value noted<br>*Give P values as exact values whenever suitable.*
[x]	[ ]	For Bayesian analysis, information on the choice of priors and Markov chain Monte Carlo settings
[x]	[ ]	For hierarchical and complex designs, identification of the appropriate level for tests and full reporting of outcomes
[x]	[ ]	Estimates of effect sizes (e.g. Cohen's d, Pearson's r), indicating how they were calculated

Our web collection on statistics for biologists contains articles on many of the points above.

Software and code

Policy information about availability of computer code

Data collection	Fluorescence and Bright-field microscopy: Instrument- Nikon Eclipse Ti2; Ziess Axio Vert al.<br>Confocal microscopy and FRAP-LMI-005-Confocal Microscope-SP8 (Leica Microsystems, Germany).<br>Light scattering: ProbeDrum lab-in-a-box S/N 318 0004.<br>Capflex measurements: FIDA 1 version 2.11.<br>The codes used in this manuscript for automated baseline analysis and Open trons OT2 pipetting robot can be found using DOI 10.11583/DTU.14223116. and DOI: 10.11583/DTU.16797556, respectively.
Data analysis	ImageJ (1.52h), Origin Pro 2019 and 2021 (academic), KaleidaGraph (v4.03), Python 3.7, Jupyter 6.1.4, ProtParam (Expasy, Switzerland).

For manuscripts utilizing custom algorithms or software that are central to the research but not yet described in published literature, software must be made available to editors and reviewers. We strongly encourage code deposition in a community repository (e.g. GitHub). See the Nature Portfolio guidelines for submitting code & software for further information.

Data

Policy information about availability of data

All manuscripts must include a data availability statement. This statement should provide the following information, where applicable:

- Accession codes, unique identifiers, or web links for publicly available datasets
- A description of any restrictions on data availability
- For clinical datasets or third party data, please ensure that the statement adheres to our policy

The authors declare that all the data supporting the findings of this study are available within the paper and in supplementary information files. All the data analysis was performed using published tools and packages and has been cited in the paper and supplementary information text. No data has been excluded. Source data

are provided with this paper.

Field-specific reporting

Please select the one below that is the best fit for your research. If you are not sure, read the appropriate sections before making your selection.

- [x] Life sciences
- [ ] Behavioural & social sciences
- [ ] Ecological, evolutionary & environmental sciences

For a reference copy of the document with all sections, see nature.com/documents/nr-reporting-summary-flat.pdf

Life sciences study design

All studies must disclose on these points even when the disclosure is negative.

Sample size	Sample size was chosen based on the reproducibility of the data from independent experiments (n=2 or more). Samples were analyzed and corresponding mean (and standard deviations wherever applicable) were calculated and reported in the main and supplementary manuscripts.
Data exclusions	No data were excluded during the analysis.
Replication	All the experiments were repeated independently at least twice/thrice. The number of replicates used in the individual experiments are mentioned in the respective figures. All the replicates were successfully reproducible and showed similar results.
Randomization	Samples were not drawn from existing distributions, but in each case produced for the specific purpose/measurement. Therefore randomization was not relevant.
Blinding	Investigators were not blinded to the studies due to the inability to administer relevant treatments during the course of the experiments if blinded.

Reporting for specific materials, systems and methods

We require information from authors about some types of materials, experimental systems and methods used in many studies. Here, indicate whether each material, system or method listed is relevant to your study. If you are not sure if a list item applies to your research, read the appropriate section before selecting a response.

Materials & experimental systems

n/a	Involved in the study	
☒	☐	Antibodies
☒	☐	Eukaryotic cell lines
☒	☐	Palaeontology and archaeology
☒	☐	Animals and other organisms
☒	☐	Human research participants
☒	☐	Clinical data
☒	☐	Dual use research of concern

Methods

n/a	Involved in the study	
☒	☐	ChIP-seq
☒	☐	Flow cytometry
☒	☐	MRI-based neuroimaging
