# Supplementary material for: Capillary flow experiments for thermodynamic and kinetic characterization of protein liquid-liquid phase separation
Source: Nat Commun. 2021 Dec 15;12:7289. doi: 10.1038/s41467-021-27433-y (PMC8674230; doi:10.1038/s41467-021-27433-y)
Supplement: Supplementary file 4 — Description of Additional Supplementary Files [file 41467_2021_27433_MOESM4_ESM.pdf]

Title: Supplementary Video 1:
Description: Fusion of Ddx4n1 liquid droplets The video shows dynamic Ddx4n1 phase separated droplets showing succesful fusion events confirming their liquid-like nature. The sample contains 100 µM of unlabeled, Ddx4n1 in 20 mM Tris pH 8.0, 100 mM NaCl, 5 mM TCEP. The sample is observed under a 40x objective (BF) and the video is captured with a speed of 1 frame/800 ms. n=2 independent experiments.

Title: Supplementary Video 2:
Description: Fusion of α-Syn liquid droplets The video shows dynamic α-Syn phase separated droplets showing succesful fusion events confirming their liquid-like nature. The sample contains 100 µM of unlabeled, WT α- Syn in presence of 20% (w/v) PEG6000 in 10 mM sodium phosphate buffer, 200 mM NaCl, pH 7.4. The sample is observed under a 40x objective (BF) and the video is captured with a speed of 1 frames/sec. n=2 independent experiments.

Title: Supplementary Video 3:
Description: α-Syn liquid droplets dissolve upon dilution The video shows dynamic α-Syn phase separated droplets (after 24 h) re-dissolving upon contact with the buffer. The data confirms that the droplets are liquid-like after 24 h. The sample contains 100 µM of unlabeled, WT α-Syn in presence of 20% (w/v) PEG6000 in 10 mM sodium phosphate buffer, 200 mM NaCl, pH 7.4. The sample is observed under a 40x objective (BF) and the video is captured with a speed of 1 frames/sec. n=2 independent experiments.
